# Crisis-Critical Intellectual Property: Findings From the COVID-19 Pandemic

**DOI:** 10.1109/TEM.2020.2996982

**Published:** 2020-06-18

**Authors:** Frank Tietze, Pratheeba Vimalnath, Leonidas Aristodemou, Jenny Molloy

**Affiliations:** Department of Engineering, Innovation and Intellectual Property Management Laboratory, Centre for Technology ManagementInstitute for ManufacturingUniversity of Cambridge 2152 CB3 0FS Cambridge U.K.; Department of Engineering, Innovation and Intellectual Property Management Laboratory, Centre for Technology Management, Institute for ManufacturingUniversity of Cambridge 145354 CB3 0FS Cambridge U.K.; Department of Chemical Engineering and Biotechnology, Open Bioeconomy LaboratoryUniversity of Cambridge 145354 CB3 0FD Cambridge U.K.

**Keywords:** Coronavirus, COVID-19, global health crisis, incumbents, innovation, intellectual property (IP), licensing, new entrants, pandemic

## Abstract

A pandemic calls for large-scale action across national and international innovation systems in order to mobilize resources for developing and manufacturing crisis-critical products efficiently and in the huge quantities needed. Nowadays, these products also include a wide range of digital innovations. Given that many responses to the pandemic are technology driven, stakeholders involved in the development and manufacturing of crisis-critical products are likely to face intellectual property (IP)-related challenges. To (governmental) decision makers, IP challenges might not appear to be of paramount urgency compared to the many undoubtedly huge operational challenges to deploy critical resources. However, if IP challenges are considered too late, they may cause delays to urgently mobilize resources effectively. Innovation stakeholders could then be reluctant to fully engage in the development and manufacturing of crisis-critical products. This article adopts an IP and innovation perspective to learn from the currently unfolding COVID-19 pandemic using secondary data, including patent data, synthesized with an IP roadmap. We focus on technical aspects related to research, development, and upscaling of capacity to manufacture crisis-critical products in the huge volumes suddenly in demand. In this article, we offer a set of contributions. We provide a structure, framework, and language for those concerned with steering clear of IP challenges to avoid delays in fighting a pandemic. We provide a reasoning why IP needs to be considered earlier rather than too late in a global health crisis. Major stakeholders we identify include 1) governments; 2) manufacturing firms owning existing crisis-critical IP (incumbents in crisis-critical sectors); 3) manufacturing firms normally not producing crisis-critical products suddenly rushing into crisis-critical sectors to support the manufacturing of crisis-critical products in the quantities that far exceed incumbents’ production capacities; and 4) voluntary grassroot initiatives that form during a pandemic, often by highly skilled engineers and scientists in order to contribute to the development and dissemination of crisis-critical products. For these major stakeholders, we draw up three scenarios, from which we identify associated IP challenges they face related to the development and manufacturing of technologies and products for 1) prevention (of spread); 2) diagnosis of infected patients; and 3) the development of treatments. This article provides a terminology to help policy and other decision makers to discuss IP considerations during pandemics. We propose a framework that visualizes changing industrial organizations and IP-associated challenges during a pandemic and derive initial principles to guide innovation and IP policy making during a pandemic. Obviously, our findings result only from observations of one ongoing pandemic and thus need to be verified further and interpreted with care.

## I. Introduction

In December 2019, an outbreak of a novel coronavirus in Wuhan, Hubei province, China, manifested itself as a global health tragedy. The World Health Organization (WHO) announced it as a public health emergency of international concern on January 30, 2020 [1] and as a pandemic on March 11, 2020 [2]. The virus, later named SARS-CoV-2 [3], can cause mild flu-like symptoms (or even be asymptotic) but can progress to acute pneumonia-like respiratory illness called novel coronavirus-infected pneumonia (NCIP). The overall clinical syndrome is known as COVID-19 [4]. Until today, there are no vaccines or medical cure for the disease yet [5], and the disease has a fatality rate that is unconfirmed due to lack of testing data for many countries but is likely to be around or above 1% [1]. In just less than six months since its emergence, the virus is affecting more than 212 countries, with more than 4 million confirmed cases worldwide [2].

The virus has a stronger transmission capacity than the “conventional” annually recurring flu. On average, without social distancing measures in place, one infected person passes the virus to 2–2.5 others (that range is subject to change and can vary largely by geography, age group, and time) [8], [9].

The current COVID-19 pandemic creates enormous demand surges for products that are crisis relevant as well as a need for rapidly developing innovations to address crisis-specific problems. Innovation efforts require pooling of and repurposing of resources, capabilities, and capacities from actors owning relevant or capable of creating new intellectual property (IP) to develop these crisis-critical innovations.

The literature that investigates IP challenges during times of global crisis appears very limited (see, e.g., [3]). A limited number of papers focus on IP challenges during economic crises, such as the global financial crisis in 2008–2009. During that crisis, strong IP protection was found to be beneficial for companies to recover, e.g., through facilitating collaboration, IP monetization, licensing, and the use of IP as collateral [4], [5]. Another small set of papers actually focuses on global health crises (see, e.g., [6]–[11]). Most authors, however, focus on crises that unfold much slower than the current COVID-19 pandemic, such as the HIV/AIDS pandemic. For ending the global HIV/AIDS pandemic, IP rights were found to be a barrier for low-income countries to access HIV/AIDS medicines after they became available [7], [12]. As a consequence, parallel import options and compulsory licensing were introduced at the international level to relax IP restrictions on essential medicines [6], [7]. Existing literature also studies compulsory licensing [6], [7], changes to patent laws, such as fast track grant procedures [6], “western subsidies” [8], restricted patentability standards, and patent pools involving voluntary nonexclusive licenses among private innovators (e.g., UNITAIDS Medicine Patent Pool) [9], [10]. While these papers undoubtedly discuss topics that are potentially relevant to the COVID-19 pandemic (compulsory licensing has already been enacted by a few countries), findings from those papers must be treated carefully and should not be overly generalized to the COVID-19 pandemic. The current pandemic spreads so much faster than the global health crises studied in prior literature. However, two general conclusions can be drawn from prior literature focusing on IP in the context of crises that are very much in line with what is known from extensive economic research on IP and innovation. First, IP seems to play a role as an innovation incentive; second, IP needs to be considered for accessing crisis-critical products (CC-P), such as vaccines and treatments. We can thus conclude that the existing literature hardly provides suitable frameworks, terminology, evidence, and guidance for (governmental) decision makers to make informed choices to best utilize IP, and to steer clear of IP associated challenges and risk during and beyond global crises.

This article aims to contribute to the many efforts to contain the pandemic as quickly as possible. We offer a set of contributions with two primary purposes. First, we hope we contribute reasoning on why IP considerations need to be addressed early rather than later during a pandemic. Second, we provide a structure (if not conceptual framework) that is hopefully helpful for those concerned with steering clear of IP challenges, e.g., policy makers, governments, international organizations, large IP owners, new entrants, and many voluntary initiatives that are part of the grassroots movement.

This article focuses on three critical areas for fighting a pandemic, all of which are technology dependent: 1) the prevention (including measures to limit its spread and vaccines to prevent future outbreak); 2) diagnosis (including professional and self-testing); and 3) treatment, with the latter including the direct treatments (e.g., development of drugs) and the treatment of symptoms, i.e., related to the medical equipment needed to keep bodies alive (e.g., ventilators and intensive care unit (ICU) beds).

Deriving findings from secondary data of the COVID-19 pandemic, including patent data, this article contributes a structure, framework, and language for those concerned with steering clear of IP challenges to avoid delays in fighting a pandemic. We identify relevant stakeholders and describe associated IP challenges they face related to the development and manufacturing of technologies and products for prevention (of spread), diagnosis of infected patients, and the development of treatments summarized in an adopted IP roadmap. Major innovation stakeholders we identify include the following:

1) governments;
2) manufacturing firms owning existing crisis-critical intellectual property (CC-IP) [incumbents in crisis-critical sectors (CC-S)];
3) manufacturing firms normally not producing CC-P suddenly rushing into CC-S to support the manufacturing of CC-P in the quantities that far exceed incumbents’ production capacities;
4) voluntary grassroot initiatives that form during a pandemic, often by highly skilled engineers and scientists in order to contribute to the development and dissemination of CC-P.

Particularly, new relationships that are formed rather suddenly during a pandemic appear to be associated with various IP related uncertainties with the particular problem that negotiating licensing agreements is typically time consuming and that new IP emerges during the pandemic, which can be owned by new entrants.

This article provides a terminology that (hopefully) supports (governmental) decision makers to discuss IP considerations during pandemics that call for urgent and large-scale actions from innovation stakeholders. We propose a framework that visualizes changing industrial organizations and IP associated challenges during a pandemic and derive initial guiding principles for innovation and IP policy making during times of a pandemic. Those can also serve as an analytical framework for others and particularly for follow-up studies. Obviously, our findings result only from observations of one ongoing pandemic and thus need to be verified further and interpreted with care.

## II. Methodology

**Fig. 1.**
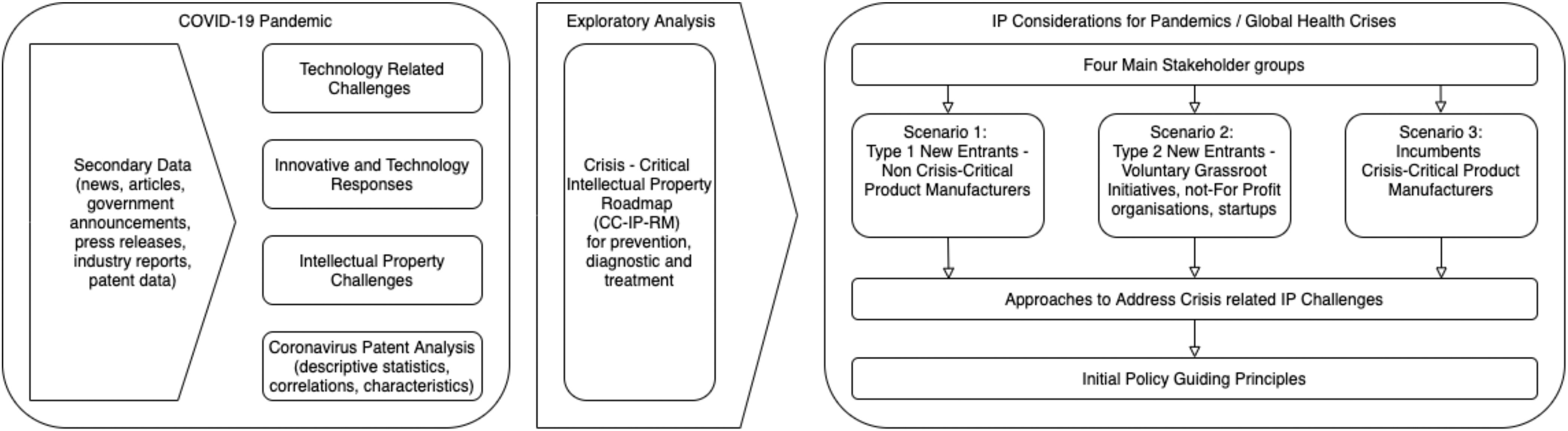
CC-IP exploratory methodology for the COVID-19 pandemic.

To contribute to filling the knowledge gap concerning IP considerations during pandemics, we deploy an exploratory method [13] employing an IP and innovation perspective (see Fig. 1). One could argue that we treat the COVID-19 pandemic as a single longitudinal case study [14], [15] to make better informed decisions during this, but also future global health crises. Our findings are based on secondary data collected during the ongoing COVID-19 pandemic. The data include publicly available documents, such as news articles, government announcements, press releases, industry reports, and patent data.

We complement our analysis of secondary data with a patent analysis for the severe acute respiratory syndrome (SARS) Coronavirus, where we make use of the open patent data sets compiled by Lens.org,^1^^1^Human Coronavirus Innovation Landscape: Patent and Research Works Open Datasets. Accessed [01.04.2020] at https://about.lens.org/COVID-19 to enhance our understanding into preventive, diagnostic, and treatment measures. We focus on the broader spectrum of coronaviruses to identify patterns from earlier outbreaks that could be applied in the case of SARS-Cov-2. We use the data set compiled by Lens.org “Coronavirus: Broad Keywords Based Patents” and extract all the related patent information.^2^^2^Dataset: Coronavirus Broad Keywords Based Patents; Patents: 6896; Patent Families: 2670; Dataset Created: 27.01.2020; Dataset Updated: 20.03.2020; Search Query: (title:(Coronavirus) OR abstract:(Coronavirus) OR claims:(Coronavirus)) OR (title:(“Severe acute Respiratory syndrome”) OR abstract:(“Severe acute Respiratory syndrome”) OR claims:(“Severe acute Respiratory syndrome”)) OR (title:(“coronaviridae”) OR abstract:(“coronaviridae”) OR claims:(“coronaviridae”)) OR claims:(“SARS-CoV”) OR claims:(“MERS-CoV”) OR claims:(“COVID 19”) OR claims:(“Wuhan coronavirus”) OR claims:(“2019-nCoV”) OR claims:(“Middle East respiratory”). We choose to focus on the keywords to capture a large variety of coronavirus-related patents, in a time of high uncertainty, to improve our overall understanding.

The COVID-19 data are then synthesized in technology-related challenges, innovative and technology responses to the COVID-19 pandemic, and IP-related challenges. Using this information, together with the patent analysis, we synthesize a crisis-critical IP roadmap to effectively provide a systematic compilation, description, and analysis of IP considerations. For that, we adapt the IP roadmapping template proposed by [16] and [17] structuring it along three time-interdependent pandemic phases: 1) prevention (reducing the spread, including vaccine development), 2) diagnosis (increase our understanding about the coronavirus and its early identification using test kits or symptom identification), and 3) treatment (treatment development of the acute respiratory pneumonia caused by COVID-19, with a preventative vision).

From this initial analysis and development of the CC-IP roadmap, we identify four main stakeholder groups. We then develop a terminology that can be used for conceptualizing IP issues during a crisis. For different stakeholder constellations, we identify and describe three scenarios. The outcomes of the scenarios lead into a CC-P/sector framework for how industrial organization can change during pandemics. We use what we have learnt from the scenarios, again using our terminology to reflect on current policy responses from different countries, which leads us to formulate initial guiding principles for IP and innovation policy making.

## III. Innovation and IP During the COVID-19 Pandemic

From an IP and innovation perspective, this section synthesizes relevant observations from the current COVID-19 pandemic. Based on publicly available secondary data, we identify technology-related pandemic challenges, describe innovative and technology responses, and finally summarize observations related to IP issues that emerge during the ongoing pandemic.

### A. Major Technical Pandemic Challenges

From our observations of the current COVID-19 pandemic, five major technology-related challenges emerge. Some relate to novel technologies highlighted in the WHO's Coordinated Global Research Roadmap for COVID-19 [18], while others emerge from operational needs in frontline healthcare.

First and foremost, the challenge of *finding a vaccine and treatments* for the acute respiratory pneumonia caused by COVID-19 has initiated large-scale R&D efforts. Second, the pandemic has created a sudden and massive demand for the *development and manufacturing of diagnostic testing kits* in extremely large volumes, not only with high accuracy that can be conducted in a high-throughput manner (e.g., for several weeks, Germany alone has carried out 160 000 tests per week [19]) but also innovative ways of organizing testing (e.g., COVID-19 isolation pods, drive through testing). Third, the pandemic caused a sudden need to treat a large number of patients in hospitals requiring an extraordinarily large ICU capacity, particularly with an enormous need for ventilator capacity (e.g., U.K. ventilator challenge [20]) by far exceeding the number currently available in many hospitals and countries, leading to supply shortages. Fourth, the COVID-19 pandemic has caused an exceptionally high demand for skilled medical staff, doctors, and nurses, particularly with ICU experience, such as anesthetists and critical care nurses, all of whom need to be equipped with personal protective equipment (PPE). Due to the high infectivity of the virus, in this pandemic, PPE such as protective clothing, face shields, goggles, and gloves are critical to protect healthcare staff from infection with SARS-CoV-2. Fifth, the pandemic created a strong need for digital innovation, including artificial intelligence (AI)-enabled tracking apps for cases and spreaders and epidemic modeling to monitor and understand the spread and development of the virus across populations.

Of course, a pandemic that cuts across all parts of life raises various other technical and nontechnical challenges. Those include, for instance, the security of supply chains for essential goods; securing of food supply with supermarket chains plays a major role, but so does the optimization of delivery route planning, quick adjustments to online booking systems, e.g., for rationing certain goods or prioritizing delivery slots to the elderly and vulnerable. Other challenges are associated with unprecedented numbers of people working from home, i.e., video conferencing platforms and equipment (e.g., Google and Microsoft announced free access to teleconferencing and collaboration tools [62]) as well as internet service providers relieving data caps. Then, there are urgent logistical challenges, for instance, to efficiently reorganize supply of CC-P in the midst of drastically reduced passenger and cargo transport routes, the repatriation of national citizens from abroad, but also internal operations processes in hospitals as wards, has been repurposed, and specific COVID-19 testing pods have been set up to innovate at health system and infrastructure level to cope with testing and treating huge numbers of people.

### B. Innovative and Technology Responses to the Pandemic

During the past weeks, we have observed a number of responses to the five technical challenges. Pharma, biotechnology firms, and universities have joined forces to develop vaccines [21] and treatments [22], also testing whether existing antiviral drugs could be repurposed, e.g., malaria/HIV drugs or development of novel COVID-19 specific drugs [23]. For instance, a collaboration of Clover and GSK has been announced [24]. Another consortium includes life sciences companies such as Novartis, Bristol Myers Squibb, and GSK [25]. Others started to develop novel diagnostics, such as BOSCH who recently announced that they developed their own COVID-19 test kit [26]. Manufacturing companies from all kinds of sectors have started to repurpose production lines to support the production of CC-P, involving large engineering/manufacturing firms such as those involved in the U.K. ventilator challenge consortium (e.g., Airbus, GKN, Roll-Royce, Siemens, and Smiths group) [20], [27], [28], and luxury brands (e.g., French LVHM) using perfume manufacturing facilities to make hand sanitizers [29]; small- and medium-sized enterprises (SMEs) have started to produce sanitizers [30]; textile manufacturers (e.g., ZARA in Spain [31], Trigema in Germany [32], and Prada in Italy [33]) are mass producing face masks. We also observe that various volunteering initiatives emerge, such as those run by scientists and engineers to develop open hardware/source designs of ventilators; develop new PPE designs, e.g., 3-D printed face shields [34] and ventilator valves [35]; and develop new ways to mass produce PPE [35], [36].

Last but certainly not least, digital innovations have sprung up widely, e.g., data/software approaches by scientists for prevention, diagnostics, and treatment. In terms of prevention, scientists have focused on developing open data platforms and epidemiological models to forecast growth curves of the virus and model the impact of government responses [37]; analyzing geospatial models to understand the distribution and spread of the virus [38]; deploying causal-effect models to understand symptoms of the virus and limit its spread through behavioral science; and tracking applications [39]. In the diagnostic sphere, scientists utilize AI and more specifically deep convolutional neural networks to detect COVID-19 from X-ray images. This has also been particularly useful in diagnostic analysis of symptoms to predict the development of a patient's case [40]. A high number of efforts have also been concentrated on treatments, where scientists have developed AI-based text and data mining tools to help the medical community to prioritize scientific questions and potential lead treatments. Efforts have focused on the development and summarization of genome-specific precision medicine based on host response, as well as on modeling and simulation of the virus propagation and efficiency of interventions [41].

From what emerged during this crisis, one can categorize the crisis-critical activities in three categories, most of them related to innovation or massive capacity building/upscaling to provide CC-P in sufficient quantity in a short period of time. The first category is *prevention*, including digital innovations to track the virus spread, sanitizers, PPE, etc., in order to slow down the spread of the virus, and vaccine developments to control future outbreaks. The second category is *diagnostics*, predominantly the need for an incredible volume of nucleic acid and antibody testing kits which are accurate, and delivering speedy results. The third category is *treatment*, including development of treatments for the acute respiratory pneumonia caused by COVID-19 through repurposing or existing drugs, development of new antiviral drugs, and ventilators for ICU critical care in hospitals around the world. We use this prevention–diagnosis–treatment framework throughout the remainder of this article.

### C. IP-Related Challenges During the Pandemic

Table I summarizes IP-related challenges (including examples) across the three phases of the prevention–diagnosis–treatment framework employing an IP roadmap structure adapted from [16] and [17]. This section provides only a brief description for some of those challenges worth highlighting particularly. Table II provides a draft overview of COVID-19-related vaccines under development.

**TABLE I:** IP Considerations for COVIE-19, Synthesized With an Adapted IP Roadmapping Framework

**COVID-19**			**Prevention**	**Diagnostics**	**Treatment**
**Why?**	**Technologies, products, services**	**Which technologies are relevant? What types are these (platform /standards)?**	Vaccine: 1) Vaccines to prevent future outbreak (see Table II for list of vaccines under development for COVID-19) [43]–[45] 2) Tools to monitor the spread of the disease [46] 3) Epidemiological models (controlling for different government responses to forecast the development of growth curves) [37] 4) Analysis of geospatial and geopolitical data of COVID19 cases [39] 5) Causal Effects and in-depth analysis of COVID19; Applying machine learning/AI methods to mitigate the spread of the COVID-19 pandemic, understanding behavioral science [47] Medical equipment/PPEs: 1) Diagnostics kits (e.g., see, [48] and [49] for list of approved COVID-19 test-kits) 2) Masks to prevent disease spread (e.g., GENTL mask, an open-source design by EPAM [50], see [51] for list of DIY masks) 3) Sanitizers to avoid spreading of disease [52]–[54] 4) Specialized products to avoid spreading of disease (e.g., Hands-free door handle attachment by 3D printing players) [55]	Medical equipment/ methods: 1) Testing kits (Self-testing kit/ Professional testing kit): 2) Home testing-kit [56] 3) Rapid testing technology [57] 4) AI tools - Detection of COVID-19 using deep convolutional neural networks from X-rays [40] 5) Diagnostic analysis and forecasting of variables of symptoms affecting patients [58]	Drugs: 1) Existing drugs that can potentially treat COVID (repurposing) - remdesivir, lopinavir and ritonavir in combination; lopinavir/ritonavir plus interferon-beta; and chloroquine and hydroxychloroquine [59] 2) Genome-specific COVID-19 medical protocols, including precision medicine of host responses 3) Modeling, simulation, prediction of COVID-19 propagation, and efficacy of interventions [58] 4) Design and sharing of clinical trials for collecting and analyzing data on medications, therapies, and interventions [47] 5) Develop AI text and data mining tools that can help the medical community develop answers to high-priority scientific questions [41] Medical equipment: 1) Ventilators, light weight portable ventilator, valves [28] [35], 2) Medical grade masks [33]
**Who and what?**	**IP ecosystem stakeholders**	**Consider the different IP ecosystem actors. Which actors are likely to play important roles?**	Incumbents from CC-Sectors: *Medical technology & tool developers:* 1) Traditional health incumbents developing test-kits (e.g., Everywell [56]) 2) Digital health community developing digital tools to monitor spread [46] 3) Pharma companies developing vaccines [24], [44], [60] *Medical and healthcare service providers:* 1) Testing service providers (telemedicine doctors) like PWNHealth [56] Universities and research institutions: 1) Universities (vaccines, sanitizers) (e.g., Oxford's vaccine [61]) New entrants from non CC-Sectors (New entrant type 1): 1) Engineering and software solution providers offering open-source designs for masks (e.g., GENTL mask, an open-source design by EPAM [50]) 2) IT giants giving free access to their platforms [62] 3) 3-D printing players developing hands-free door handle attachments [55]	Incumbents from CC-Sectors: *Medical technology & tool developers:* 1) CC-S incumbents 2) Universities (E.g., Oxford's rapid testing technology) [57] *Medical and healthcare service providers:* 1) Testing service providers (telemedicine doctors) like PWNHealth [56]	Incumbents from CC-Sectors: *Drug developers:* 1) Big pharmaceutical companies: GSK, Novartis, Bristol Myers Squibb, AbbVie [25] *Medical technology & tool developers:* 1) Digital health community developing tools to help treatment [46] Universities: 1) University start-ups make ventilators (e.g., University of Toronto) [63] New entrants from non - CC s ectors : 1) 3-D printing companies with ventilators/valves [35] 2) Aerospace/automotive industry in ventilators [20], [27], [28] 3) Household appliances industry (e.g., Dyson's CoVent applying Dyson's air purifier expertise to develop ventilators) [64] 4) Fashion industry to make masks (e.g., Zara) [31], Tirgema [32], Italy [33])
			Start-ups from CC s ectors (New entrant type 2): Start-ups developing supportive testing solutions & partnering with medical centers (e.g., blood testing by Sight Diagnostics, Israel, start-up with Sheba Medical Center) [65] New entrants from non - CC s ectors (New entrant type 1) Publishers providing free access to research and technologies (e.g., Springer Nature, PubMed Central, Association of American Publishers, and IEEE [66]–[68]) Platform creators/integrators: Platforms to pledge IP (e.g., Open COVID pledge) [69]–[71] Charity organizations: Research charity organizations and foundations (e.g., Wellcomme trust and Billand Melinda Gates Foundation [72]) Government bodies: 1) WHO (e.g., provides standard for sanitizers, asked to create patent pools); 2) National governments (announcing and considering compulsory licensing) [59]; 3) Government-industry engagements (e.g., Johnson & Johnson expanded an existing R&D agreement with the US Department of Health and Human Services (HHS) for COVID-19 vaccine development) [73]		
	**IP purpose**	**What it serves?**	1) Addressing supply shortages of Masks. Free design/DIYs free up the demand for clinical grade masks for use in hospitals [51] 2) Access to critical and relevant knowledge and social awareness through copyrights waiver and offering free contents [66]–[68] 3) Accelerating vaccine development through consortiums and pooling expertise [74]	Accelerated development and wider availability of testing kits through IP sharing [35]	1) Wider accessibility of medicines through access to patents (e.g., AbbVie has agreed to drop enforcement of Kaletra patents) [75] 2) Addressing supply shortages in ventilators [28] 3) Accelerated development for ventilators [76]
		**IP Challenges**	IP imitation and risk of counterfeit products	1) Patent infringement lawsuits attempts delaying development of testing by existing players (e.g., lawsuit by Labrador Diagnostics LLC against BioFire) [77]	1) Declining access to existing CC-IP may lead to reverse - engineering by new entrants from non CC-S (e.g., Italian volunteers 3-D print reverse-engineered valves after denial of IP) [35]. 2) Patents as barriers to the production and provision of low-priced treatments [59]
			Monopoly over CC-IP may increase cost of the COVID-19-related medicines and in turn the price. Proactive measures by governments is the implementation of compulsory licensing [59]		
**How?**	**IP assets and strategies**	**IP Assets**	1) Designs (DIY masks, hands-free door handle attachment) [51] [55] 2) Patents (e.g., vaccination related)	1) Patents for testing technology 2) Designs for test-kits	Patents (e.g., drug related patents including product and process patents, patents of technologies for medical equipment like ventilators, valves) New patents (e.g., Isinnova plans to patent Charlotte Valve) [35]
			Copyrights—free copyright (open access articles) provides access to relevant, knowledge, and research which would otherwise be not available [66]–[68]		
		**Which IP strategies are best suited to help achieve the IP purpose?**	1) Open-source design for masks. (e.g., GENTL mask, an open-source design by EPAM [50]) 2) Free access for limited time (e.g., IT giants Google & Microsoft providing free access to their conference tools to support remote working) [62] 3) Bilateral collaborations for vaccine development (e.g., Sanofi with Translate Bio; GSK with Clover)[24], [78] 4) Consortiums for vaccine R&D [74]	1) Royalty free licensing of patent-protected diagnostics technology (e.g., Labrador Diagnostics, a subsidiary of Fortress Investment Group)[79]	Compulsory licensing: 1) Compulsory licensing for COVID-19 drugs (e.g., Canada, Chile) [59] Free access: 2) Obtaining new patent protection and giving the patent free for others to use [80]; free access and usage rights to existing patents [75] [81] Multi sector collaboration: 3) Multisector consortiums to accelerate ventilator production to address demand–supply shortage, e.g., ventilator Challenge U.K. [28] 4) R&D in Non-CC sectors to apply expertise from non-CC sectors to develop medical equipment (e.g., Dyson's CoVent) [64]
			1) Voluntary pool by government bodies (e.g., WHO asked to create voluntary pools [82]) 2) Consortiums among big pharma companies to accelerate the development, manufacture, and delivery of vaccines, diagnostics, and treatments for COVID-19' [83] 3) Pledge to give free access to all IP for limited time. Voluntarily or through third-party platforms (e.g., Open COVID pledge) [69], [70] 4) Hackathons to share and generate ideas [84] 5) Open Platform Sharing of Datasets and Source Code [85]

**TABLE II:** Vaccine Development Sample, Total of 54 Vaccines (Two in Phase 1 Clinical Trials and 52 in Preclinical)^*^

**Platform**			**Type of candidate vaccine**	**Developer**	**Clinical evaluation stage**	**Platform (non-coronavirus)**
**1**		Nonreplicating Viral Vector	Adenovirus Type 5 Vector	CanSino Biological Inc. and Beijing Institute of Biotechnology	Phase 1; ChiCTR2000030906	Ebola
**2**		RNA	LNP-encapsulated mRNA	Moderna/NIAID	Phase 1; NCT04283461	multiple candidates
**3**		DNA	DNA plasmid vaccine Electroporation device	Inovio Pharmaceuticals	Preclinical	Lassa, Nipah, HIV, Filovirus, HPV, Cancer indications, Zika, Hepatitis B
**4**		DNA	DNA	Takis/Applied DNA Sciences/Evvivax	Preclinical	
**5**		DNA	DNA plasmid vaccine	Zydus Cadila	Preclinical	-
**6**		Inactivated	Inactivated + alum	Sinovac	Preclinical	SARS
**7**		Inactivated	Inactivated	Beijing Institute of Biological Products/Wuhan Institute of Biological Products	Preclinical	-
**8**		Live Attenuated Virus	Deoptimized live attenuated vaccines	Codagenix/Serum Institute of India	Preclinical	HAV, InfA, ZIKV, FMD, SIV, RSV, DENV
**9**		Nonreplicating Viral Vector	MVA-encoded VLP	GeoVax/BravoVax	Preclinical	LASV, EBOV, MARV, HIV
**10**		Nonreplicating Viral Vector	Ad26 (alone or with MVA boost)	Janssen Pharmaceutical Companies	Preclinical	Ebola, HIV, RSV
Note: ^*^DRAFT landscape of COVID-19 candidate vaccines, URL: https://www.who.int/blueprint/priority-diseases/key-action/Novel_Coronavirus_Landscape_nCoV_Mar26.PDF, last accessed Mar. 26, 2020.						

*Note:* *DRAFT landscape of COVID-19 candidate vaccines, URL: https://www.who.int/blueprint/priority-diseases/key-action/Novel_Coronavirus_Landscape_nCoV_Mar26.PDF, last accessed Mar. 26, 2020.

IP issues hardly surfaced during the early stages of this pandemic but have been the focus of several initiatives more recently. The Wellcome Trust appears to be among the first prominent organizations that understood the relevance of IP for this pandemic [67], [109]. On January 31, 2020, the Trust called for journals, publishers, etc., to allow widespread sharing of all potentially relevant research and data set. This initiative is geared toward encouraging publishers to not to put any COVID-19 relevant publications behind a paywall. The pledge seems to be a huge success as a wide range of renowned organizations have signed up, including leading journals, such as *Nature* and *The Lancet*, the European Commission, publishers (e.g., Cambridge University Press), national academies of science (e.g., Academy of Medical Sciences, The Royal Society), foundations (e.g., Bill & Melinda Gates Foundation), research councils (e.g., Medical Research Council), ministries (e.g., Indian Department of Biotechnology, Ministry of Science and Technology), and a wide range of other organizations, including companies (e.g., BenevolentAI and Johnson & Johnson). By now, more than 24 000 research papers are available online [66]. In the past weeks, some other organizations have started raising awareness that IP might become an issue during the pandemic and have called for the government and private sector to respond. For instance, on March 27, 2020, Doctors Without Borders publicly announced their concern that firms might try to profiteer from the crisis [113] and the government of Costa Rica called the WHO to organize the pooling or pledging of IP [82]. By now, a few governments have passed compulsory license resolutions for CC-IP, e.g., Chile and Canada [59], [101]–[104], and some even authorized issuance of compulsory license, e.g., Israel's compulsory licensing for Kaletra [103].

We have also observed some initial approaches that firms have taken related to IP during this pandemic. Some companies have taken steps against the risk of counterfeit products being distributed in the crisis, e.g., PPE masks [114], [115]. Some companies enforced IP lawsuits against other companies developing CC-P. For instance, Labrador Diagnostics LLC sued BioFire, a company developing COVID-19 testing kits for infringing two of its patents [77] but later announced a royalty-free licensing to anyone developing COVID-19 tests [79]. Some firms have already filed patents or other forms of exclusivity, e.g., Gilead applied for “orphan drug” designation for potential COVID-19 treatment Remdesivir [116], [117] but dropped the application a few days later after public criticism [116].

Very recently, we have seen a limited number of firms adopting at least some selective open IP approaches, particularly pledging [105] relevant IP, such as Fortress [79], AbbVie [75], and medical device companies manufacturing ventilators (including design specifications and files), such as Irish Medtronic [107] and U.K.-based Smiths Group [108], and even individuals. A recent initiative of scientists and lawyers has also launched the Open COVID Pledge (www.opencovidpledge.org) calling IP owners to not assert relevant IP during the crisis defined until one year after the WHO declares the pandemic to be over [69]–[71]. Some of the world's largest patent owners have joint the Open COVID Pledge, such as IBM, Microsoft, and Intel. Very recently, the Open COVID Pledge has joined forces with a similar Asian initiative from Japan, to which companies such as Canon and Toyota have signed up [42].

### D. Coronavirus Patent Landscape

**Fig. 2.**
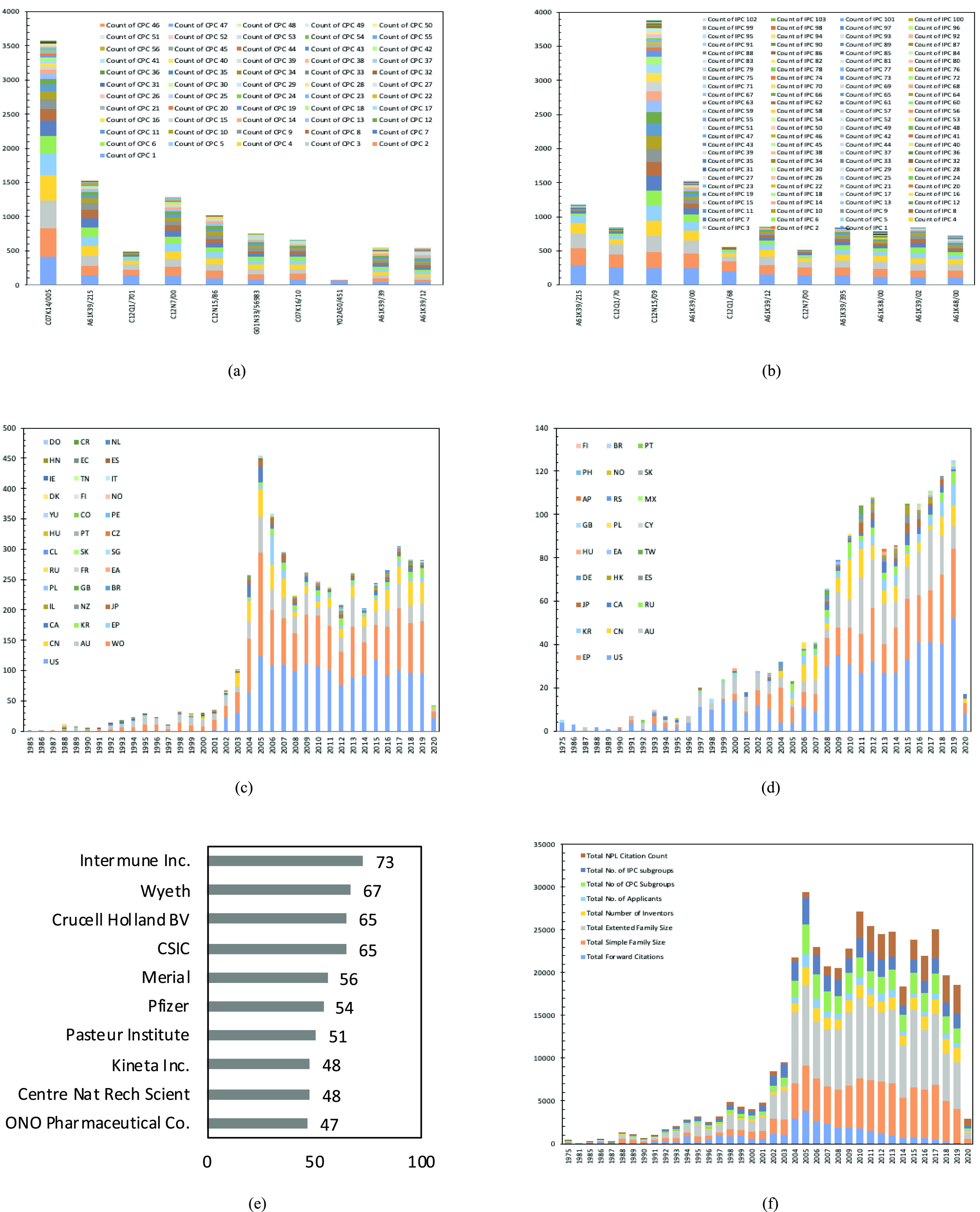
Results from the coronavirus patent analysis. (a) Top ten CPC classification distribution (sorted by descending order of primary CPC main group). (b) Top ten IPC classification distribution (sorted by descending order of primary IPC main group). (c) Patent applications distribution versus publication year (filter by jurisdiction). (d) Granted patents distribution versus publication year filter by jurisdiction. (e) Top ten applicants (co-applications are also included in the above data). (f) Distribution of patent characteristics versus the publication year.

One of the main IP challenges, both in this pandemic and in general, is the availability of open data for analyzing the progression of the virus [86], as well as the different analysis types deployed [87]. In an outbreak as severe as the COVID-19, where the reported cases have grown exponentially from around 1 million at the beginning of April 2020 to more than 4 million in just six weeks, any available data set is potentially helpful to derive insights into the disease. This section reports results from a patent analysis (see method section for details on the data set, provided by Lens.org^3^^3^Human Coronavirus Innovation Landscape: Patent and Research Works Open Datasets. Accessed on Apr. 1, 2020. [Online]. Available: https://about.lens.org/COVID-19). Table III shows the descriptive statistics and correlations for the patent data set, and Fig. 2 shows some initial results from the Coronavirus Patent Analysis. Table III reveals that the mean publication year is 2010, with a median of 2011, and a mode of 2005, which is also supported by Fig. 2(c). The mean number of applicants is 2, and the number of inventors is approximately 4. It is important to note here the large number of forward citations, with the mean around five citations and the max at 555. We find the expected behavior about forward citations, with a positively skewed distribution representing the technological impact of patent [88]. Also, worth mentioning is the fact that these patents have a large mean simple family size of approximately 14 members and a mean extended family size of approximately 21 members.

**TABLE III:** Descriptive Statistics and Correlations for Coronavirus: Broad Keyword-Based Patents (Patents = 6896 and Patent Families = 2670)^4^

	**Publication Year**	**Application year**	**Earliest priority year**	**Number of applicants**	**Number of inventors**	**Forward citations**	**Simple family size**	**Extended family size**	**Sequence count**	**Number of CPC subgroups**	**Number of IPC subgroups**	**NPL citation count**
**Mean**	2010	2008	2007	1.99	3.78	4.78	13.81	20.58	465.78	5.97	5.88	5.62
**Std. Error**	0.08	0.08	0.08	0.02	0.03	0.20	0.17	0.57	156.48	0.06	0.08	0.23
**Median**	2011	2008	2006	1.00	3.00	0.00	9.00	11.00	0.00	3.00	4.00	0.00
**Mode**	2005	2004	2003	1.00	2.00	0.00	2.00	2.00	0.00	3.00	3.00	0.00
**Std. Dev.**	6.33	6.55	6.53	1.99	2.48	16.81	14.18	47.58	12994	5.20	6.20	19.27
**Kurtosis**	1.09	0.93	0.93	13.67	7.01	313.38	4.12	318.23	1354.76	9.65	19.00	66.04
**Skewness**	-0.78	-0.65	-0.60	3.10	2.30	13.22	1.87	14.88	35.86	2.60	3.22	6.76
**Min.**	1975	1973	1972	0.00	2.00	0.00	1.00	1.00	0.00	3.00	0.00	0.00
**Max.**	2020	2020	2020	18.00	22.00	555.00	117.00	1173.00	544 026.00	56.00	103.00	342.00
**Correlations**												
**Publication year**	1											
**Application year**	0.94	1										
**Earliest priority year**	0.91	0.96	1									
**Number of applicants**	-0.09	-0.04	-0.04	1								
**Number of inventors**	0.12	0.12	0.13	0.36	1							
**Forward citations**	-0.21	-0.18	-0.17	0.12	0.00	1						
**Simple family size**	-0.01	-0.08	-0.15	-0.05	-0.01	0.03	1					
**Extended family size**	-0.05	-0.07	-0.11	0.03	0.03	0.12	0.34	1				
**Sequence count**	0.02	0.02	0.03	0.03	0.01	0.00	-0.02	-0.01	1			
**Number of CPC subgroups**	-0.10	-0.10	-0.13	0.12	0.05	0.14	0.00	0.05	0.04	1		
**Number of IPC subgroups**	-0.21	-0.26	-0.29	-0.02	0.00	0.07	0.26	0.12	-0.01	0.20	1	
**NPL citation count**	0.10	0.08	0.05	0.07	0.04	0.10	0.04	0.05	0.00	0.05	0.00	1
Note: Search Query: (title:(Coronavirus) OR abstract:(Coronavirus) OR claims:(Coronavirus)) OR (title:(“Severe acute Respiratory syndrome”) OR abstract:(“Severe acute Respiratory syndrome”) OR claims:(“Severe acute Respiratory syndrome”)) OR (title:(“coronaviridae”) OR abstract:(“coronaviridae”) OR claims:(“coronaviridae”)) OR claims:(“SARS-CoV”) OR claims:(“MERS-CoV”) OR claims:(“COVID 19”) OR claims:(“Wuhan coronavirus”) OR claims:(“2019-nCoV”) OR claims:(“Middle East respiratory”).												

*Note:* Search Query: (title:(Coronavirus) OR abstract:(Coronavirus) OR claims:(Coronavirus)) OR (title:("Severe acute Respiratory syndrome") OR abstract:("Severe acute Respiratory syndrome") OR claims:("Severe acute Respiratory syndrome")) OR (title:("coronaviridae") OR abstract:("coronaviridae") OR claims:("coronaviridae")) OR claims:("SARS-CoV") OR claims:("MERS-CoV") OR claims:("COVID 19") OR claims:("Wuhan coronavirus") OR claims:("2019-nCoV") OR claims:("Middle East respiratory").

Fig. 2(a) shows the top ten cooperative patent classification (CPC) classification distribution at the subgroup level. It is evident that the highest number of patents belonging to the primary CPC subgroup classification is in C07K14/005. This is the organic chemistry subclass, for peptides with more than 20 amino acids and specifically for viruses, which constitute viral proteins. This is followed by subgroup A61K39/215 (medicinal preparations containing antigens or antibodies materials for immunoassay for coronaviridae) and C12Q1/701 (measuring or testing processes involving enzymes, nucleic acids, or microorganisms involving virus-specific hybridization probes). These are followed by C12N7/00 (preparation of viruses bacteriophages compositions, medicinal viral antigen, or antibody compositions), G01N1 covering investigation processes for measuring or testing other than immunoassay, involving enzymes, and C07K16/08 for investigation of immunoglobulins from RNA viruses. Interestingly, within the top ten, we also find section Y (Emerging Cross-Sectional Technology), with Y02A50/451, which is specific for genetic or molecular screening of pathogens. This indicates that within the granted patents, there are some vaccination patents available. All the top ten CPC classifications have to do specifically with the chemical characteristics of the virus for prevention, diagnostics, and treatment. Also, one could note the granularity of patent applications in these fields since some of these have 50 CPC subgroups.

Fig. 2(b) shows the top ten IPC classification distribution at the subgroup level. There are 103 unique subgroups referenced on these patents. While the highest primary IPC sub group can be found at A61K39/215 (preparation for medical purposes devices or methods specially adapted for bringing pharmaceutical products into particular physical or administering forms chemical aspects of, or use of materials for deodorization of air, for disinfection or sterilization, or for bandages, dressings, absorbent pads, or surgical articles soap compositions for coronaviridae), the highest collective number is for subgroup C12N15/09 (microorganisms or enzymes compositions thereof propagating, preserving, or maintaining microorganism mutation or genetic engineering culture media microbiological testing media recombinant DNA technology).

Moreover, 64% of the top ten IPC subgroups fall within the A61K subclass [89], which covers the following subject matter under a mixture composition or process of preparing a composition or treating process: drug or other biological compositions, which are capable of preventing, alleviating, treating, or curing abnormal or pathological conditions of the living body by such means as destroying a parasitic organism or limiting the effect of the disease or abnormality by chemically altering the physiology of the host or parasite (biocides); maintaining, increasing, decreasing, limiting, or destroying a physiological body function; diagnosing a physiological condition or state by an *in vivo* test; and *in vitro* testing of biological material. Therapeutic activity of medicinal preparations is further classified in subclass. The rest of subclasses fall within the C12 class, where viruses, undifferentiated human, animal, or plant cells, protozoa, tissues, and unicellular algae are considered as microorganisms. From Fig. 2(a) and (b), it is evident that the highest cluster of patents are around the chemical process of identification, composition, and vaccine development of the coronavirus family.

Fig. 2(c) shows the distribution of patent applications within the different jurisdictions. Fig. 2(d) shows the distribution of the granted patents within the different jurisdictions. Immediately, it is evident that the patent applications are lacking behind the granting process, as there are significantly more patents applied for than granted. There are some old granted patents that fall within the broad spectrum of the analysis in 1975 about the development of vaccines, which is the reason why a patent application prior to 1975 is not found. The majority of patent applications are filed in the U.S., followed by the transition to WO under the Patent Cooperation Treaty. These are followed by AU, CN, EP, CA, KO, and JP.

The distribution of granted patents follows an upward trend (greater rate of increase than that of patent^4^^4^Human Coronavirus Innovation Landscape: Patent and Research Works Open Datasets. Accessed on Apr. 1, 2020. [Online]. Available: https://about.lens.org/COVID-19 applications), but with significantly lower numbers [with the peak around 120 granted patents in 2019 relative to an average of 300 patent applications from Fig. 2(c)]. The distributions of these granted patents within the different jurisdictions are US, European Patent Office (EP), Australia (AU), China (CN), Korea (KR), Russia (RU), and Japan (JP). Also, it is important to note that the peak distribution of patent applications around this broad category is close to the virus outbreaks. The SARS-CoV-1 outbreak originated in China in 2002, and from Fig. 2(c), we can see an exponential growth in patent applications reaching a peak in 2005. The MERS-CoV outbreak originated in Saudi Arabia in 2012, and again, there is an exponential growth of patent applications from Fig. 2(c), reaching a peak in 2017 (this was a weaker signal than the first outbreak). A more in-depth analysis reveals that the earliest priority year peak is 2003 for SARS-CoV-1 and 2015 for MERS-CoV. Therefore, we can conclude that medical efforts take at least one year to materialize in patents and then with a time lag of three to four years to be published by the patent offices.

Fig. 2(e) shows the top ten applicants in the specific field with Intermute Inc., having the highest number of patents. One observation is that the top ten applicants in the field are close to each other, within 50–70 patents, and there appears to be no monopoly. Moreover, Fig. 2(f) shows an overall distribution of different patent characteristics against the publication date. One observation is that later patents tend to ground the work more in the academic literature, evident by the increase in the total citation count of nonpatent literature (NPL). This can also be the case when at the point of an outbreak, the community makes findings available quickly with an open IP approach, e.g., via the archive publication method or publishing via journals, to make the information available and increase its effectiveness. In addition, there is an increase in the size of simple patent families and extended patent families, which indicates the wider filing strategies, and the importance of this type of patents for managing and providing diagnostics/treatment for pandemic outbreaks for the world.

## IV. IP Considerations During Pandemics

**Fig. 3.**
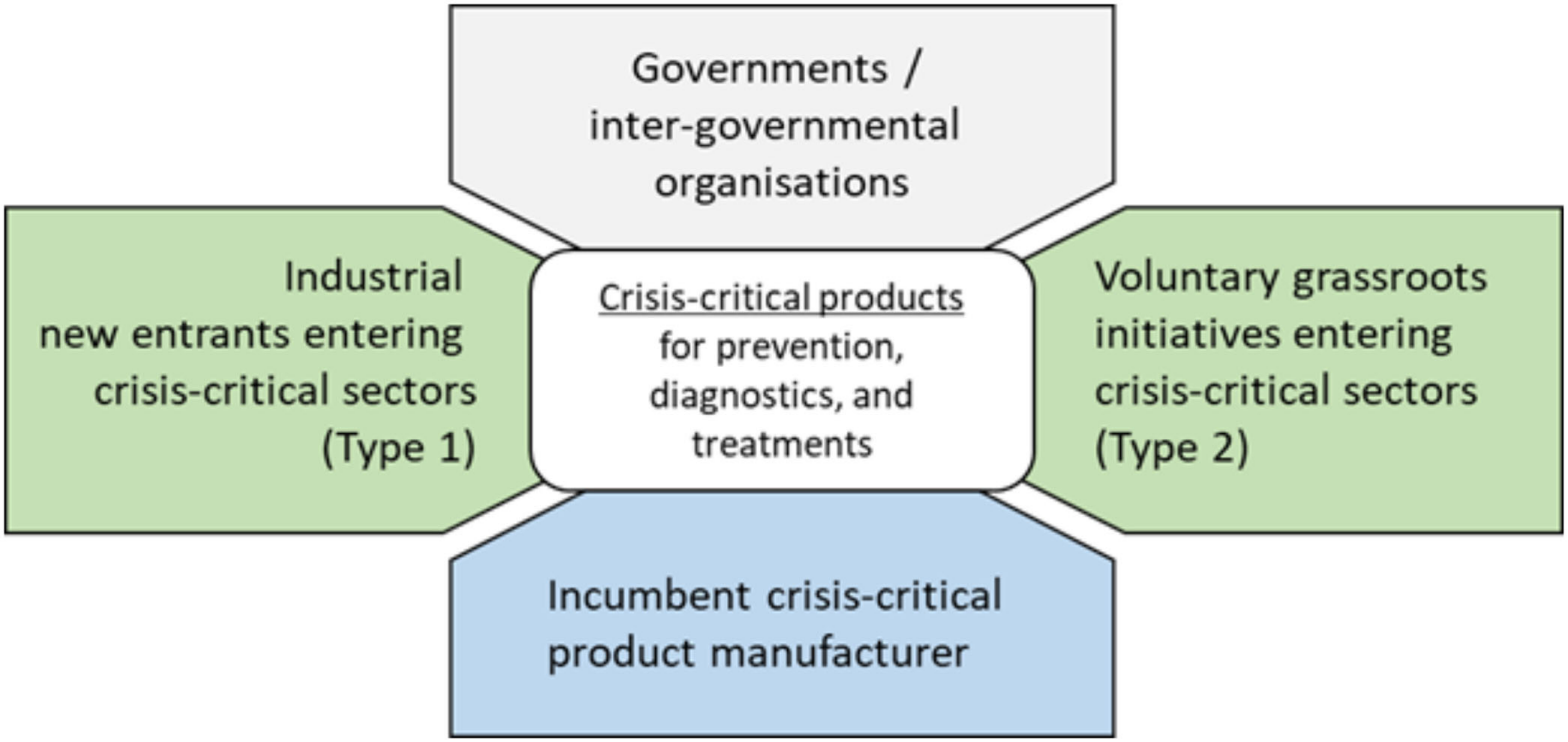
Four main stakeholder groups that are concerned with IP during a pandemic.

In order to derive findings from the observations collected from the COVID-19 pandemic, we first provide a crisis-specific terminology for discussing IP challenges during pandemics. The introduction of that terminology is then followed by a discussion of three scenarios that illustrate IP challenges during pandemics involving different constellations of the four main stakeholder groups shown in Fig. 3. Following that, we provide a framework for conceptualizing changing industrial organizations during pandemics, followed by an initial discussion of policy responses including some first thoughts about guiding principles for IP and innovation policy making.

### A. Terminology for Considering IP During Pandemics

The urgency of responding to a global pandemic has unleashed a plethora of efforts worldwide to tackle this pandemic as quickly as possible. Healthcare remains the utmost priority. Among other activities, research, technology, and innovation efforts are pouring in to support healthcare systems through development and manufacturing scale-up of what we label as CC-P, e.g., vaccines, PPE, diagnostics tests, treatments, ventilators, etc. While this calls for existing manufacturers (incumbents) that operate in what we call CC-S to quickly ramp up their production capacities, it also draws various other organizations into CC-S that have not produced CC-P prior to the pandemic. These “new entrants” include large manufacturing firms from more or less related sectors (Type 1 entrants), as well as voluntary initiatives, not-for-profit organizations, scientists, start-ups, and other forms of grassroots initiatives (Type 2 entrants).

We define CC-IP as the set of IP, relevant for the research, development, manufacturing, and distribution for CC-P, services, and technologies that are urgently needed for quickly ending a crisis situation, with crisis defined as a situation, which threatens the human species and where an approved World Body elevates the risk to be critical, i.e., in the case of COVID-19, the WHO has declared this situation a pandemic. CC-IP includes the following: 1) formal and registered IP, such as patents, design rights, and trademarks; 2) formal unregistered IP, e.g., copyrights, design drawings, CAD files; and 3) informal IP, e.g., trade secrets for manufacturing processes. CC-IP refers both to already existing (background) IP predominantly owned by incumbents that already operate in CC-S prior to a pandemic and novel (foreground) CC-IP that is developed during a crisis by various kinds of actors, including incumbents, and new (Type 1 and 2) entrants to CC-S.

Certain CC-S and their related technologies, products, and services have different degrees of formal and informal IP. Some of these products are more “high-tech” than others. Previous studies show that IP appears to be of particular importance in certain sectors, such as electronics, ICT, high-value manufacturing, software, pharmaceutical, biotechnology, medical devices, and life sciences [90], [91]. Many of those IP intensive sectors are of particular relevance in the currently unfolding pandemic. For instance, ventilators are typically expensive medical devices, and the incumbent manufacturing companies are likely to own alive/active (i.e., not yet expired) CC-IP. In contrast, PPE (e.g., face masks)—with some innovative exceptions, e.g., 3M—are fairly “low tech,” with a high probability that formally relevant CC-IP has expired.

When sudden dynamics alter industrial organizations, various actors find themselves engaged in new relationships to jointly develop and solve manufacturing challenges. These open innovation efforts involve different combinations of incumbents and new entrants (Types 1 and 2), which are likewise concerned about IP, but face different IP-related challenges. We explore those challenges for different stakeholders in the following scenarios. We use this terminology for the following scenarios and the remainder of this article with the hope that these notions serve as a “language” for others to discuss IP and innovation during times of crises.

### B. IP Scenarios During Pandemics

The following conceptual scenarios are built on the distinction between major stakeholder groups. First, “incumbents” are firms that already operated in CC-S before the start of the pandemic developing, manufacturing, and supplying CC-P. Incumbents typically own CC-IP when a pandemic starts. Second, “new entrants” are organizations that rush to enter CC-S after the beginning of a pandemic in order to support the development and manufacturing scale-up of CC-P. New entrants can be industrial manufacturing firms (Type 1) and voluntary grassroot initiatives, including not-for-profit communities, start-ups, entrepreneurial scientists, etc. (Type 2). While manufacturing firms entering CC-S could own IP, this might not be particularly relevant CC-IP. In contrast, voluntary grassroot initiatives typically do not own any formal IP prior to a pandemic as they usually only form during a pandemic. Based on the distinction between Type 1 and 2 entrants, we discuss two broad scenarios, followed by a specific scenario focused on IP challenges for incumbents.

#### 1) Scenario 1. IP Considerations for Type 1 Entrants—Non-CC-Product Manufacturers

Existing often large manufacturing firms that did not produce CC-P before the start of the pandemic are 1) either called in (ordered by governments) to help with upscaling the production of CC-P (e.g., U.K. government's call for firms to produce ventilators [20], [92]) or 2) voluntarily switch their production to CC-P, e.g., because their usual products are not in demand anymore during a pandemic (e.g., luxury company LVHM starting to produce healthcare products such as sanitizers, hydroalcoholic gel [93]). We consider those firms as Type 1 entrants.

There appears to be four groups of Type 1 new entrants. The first group includes companies that have spare manufacturing capacity as well as a set of valuable resources and capabilities that are somewhat related and can be readily used with a minimal change for production of certain CC-P to meet the supply shortage. Examples include companies such as LVHM, which had been producing perfumes before the COVID-19 pandemic, so own manufacturing process equipment to fill bottles with alcohol-containing liquid. During the pandemic, they changed the liquid from perfume to sanitizer.

The second group includes tech giants that possess diverse capabilities and rich resources that they can deploy to basically manufacture any product based on the IP from the incumbent in the CC-S. Examples include automotive companies entering the production of ventilators (e.g., Volkswagen [94]) and multinationals such as BOSCH starting to produce diagnostic test kits [26].

The third group of Type 1 entrants includes firms that possess relevant expertise, resources, and competence that can be put to use for developing and manufacturing CC-P to address a shortage of certain CC-P. An example is the U.K. company Dyson, which has long been in the air-flow business for its vacuum cleaners and hair dryers, has expertise in air purification technologies with some of its technologies such as its digital motor already optimized for safety and efficiency. Dyson, hence, was able to design a ventilator of its own called CoVent in just ten days [64]. Another example is GENTL masks, an open-source design for masks by EPAM, an engineering and software solution provider and platform developer [50].

The fourth group includes companies that possess a set of particular skills, so they can be extremely agile to speedily develop any kind of complex product, often possessing flexible manufacturing facilities and equipment, but are often limited to low volume production. Examples include formula one teams, such as Williams Racing and McLaren that joined the U.K. ventilator challenge consortium [20].

All of these four Type 1 entrants typically own formal IP prior to the beginning of a pandemic. These firms, being mostly large, commonly operate their own in-house IP departments and, thus, possess awareness of IP relevance and understanding how IP functions. However, their own IP might not be exactly relevant in the CC-S for CC-P. When entering CC-S to support the scaling up of CC-P manufacturing, these new entrants need to understand quickly how they can start manufacturing CC-P in large volume. Essentially, to do this, they can adopt three strategies.

A first strategy is not to worry (and/or care) about incumbent's CC-IP when denied access to proprietary information and know-how and go ahead, thus (willfully) infringing existing CC-IP by reengineering incumbent CC-P, e.g., Italian 3-D printing volunteers were denied access to proprietary information about ventilator valves forcing them to reverse-engineer the design [35]. In some cases, incumbents enforce their IP and pursue the new entrants for infringement. So far, this has been rare, e.g., the case of Labrador Diagnostics LLC as previously mentioned, which is understandable during a pandemic due to potential reputational damages. However, new entrants choosing this option might become future targets for infringement claims from incumbents once the pandemic has ended. While Type 1 new entrants are often large firms, which can repurpose large manufacturing volumes, well-established SMEs also enter the production of CC-P, e.g., local distilleries starting to produce sanitizers [30], [52] and 3-D printing companies producing PPE [95]. Given their small scale, they might not be particularly prone to be attacked by incumbent IP owners, but they would be in a vulnerable position if that situation did arise.

A second strategy that Type 1 entrants can adopt is to start designing CC-P from scratch, possibly using their own engineering design competence and ability to procure rapid expert advice. For example, Dyson's ventilator design Covent was developed in partnership with The Technology Partnership [64]. Other efforts are being led by coalitions of large technology consultancies such as Cambridge Consultants, who typically work on medical device projects for clients but without holding their own IP [96]. However, starting from the ground up might not be the most efficient way to achieve impact during a pandemic as developing and obtaining medical approval for new CC-P designs is likely to cause further delays. Adopting this strategy, new entrants would essentially develop novel CC-IP. However, if not carrying out careful freedom to operate analysis, they may infringe on existing background CC-IP owned by incumbents. Carrying out freedom to operate analysis may further delay the quick manufacturing of CC-P, so new entrants are left balancing these risks under uncertainty and rapid changes to the technological, regulatory, economic, and legal landscape.

A third strategy is to access CC-IP through teaming up with incumbents to produce some existing CC-P manufactured prior to the pandemic by the incumbents only. For instance, the Smiths group and Penlon are incumbents with their own ventilator designs who joined the U.K. ventilator consortium [97]. These companies can grant licenses and share CC-IP with consortium partners, including new entrants. Other incumbents, e.g., Medtronic, selectively pledged CC-IP for their Puritan BennettTM 560—a basic ventilator model for which they have made available all designs and manufacturing details under a permissive license for a limited term [81]. For new entrants, to adopt pledged CC-IP is a way to avoid infringing CC-IP owned by incumbents. For incumbents, to pledge CC-IP is a way to facilitate the adoption of their technology during the pandemic, potentially with some lasting benefits beyond the crisis. For instance, they can share CC-IP during a pandemic freely without charging any royalties using licenses that are time limited. If companies want to continue using that IP beyond the pandemic, the licensing terms would either prevent that or these companies would have to pay royalties.

Whether new entrants start developing designs for CC-P from scratch, license existing CC-IP, or infringe upon existing CC-IP, when scaling up production using their own resources, Type 1 entrants are likely to develop novel (foreground) CC-IP. Given that they are faced with resource constraints having to manufacture CC-P with equipment at their disposal and with materials they can access quickly through existing supplier relationships, they may well end up adapting existing designs. This may lead them to find inventive designs or ways to, e.g., manufacture CC-P in a cheaper way. New entrants could possibly consider formally registering this new CC-IP.

Suddenly, incumbents may find themselves confronted with new entrants in “their” sector which infringe their CC-IP, which they find difficult to enforce during a pandemic, with new entrants developing subsequently their own CC-IP. Incumbents may fear that some of the new entrants continue to stay in “their” sectors, i.e., CC-S even after the pandemic eventually ends. Having established capabilities to manufacture CC-P in innovative ways of using innovative designs, Type 1 entrants may have few incentives to stop producing CC-P and exit CC-S. Incumbents may find themselves having helped to establish new competitors by not enforcing CC-IP. The extent to which this is a risk will depend on the incumbent's existing business model and IP strategy and their interaction with new entrants; therefore, it is difficult to predict at present the severity of concern or whether it is justified.

#### 2) Scenario 2. IP Considerations for Type 2 entrants—Voluntary Grassroot Initiatives, Not-For-Profit Organizations, and Start-Ups

During the past weeks, we observed a large number of newly launched voluntary grassroots initiatives, not-for-profit organizations, and start-ups joining the development and production of CC-P. These initiatives often adopt explicitly or implicitly open-source approaches widely sharing their designs. It seems we can distinguish two groups of such initiatives.

First, we have seen that a number of highly innovative voluntary initiatives got active to help with developing and manufacturing CC-P. Those are typically founded by highly skilled people, such as engineers and scientists, and often develop fairly complex (high-tech) CC-P, including hardware such as ventilators, and data platforms to collate pandemic data or tracking applications. For instance, a large number of institutions have developed complex epidemiological models and geospatial models to understand the spread and development of the virus together with behavior science and to discuss the different approaches that have been used by governments (nonmedical interventions) to slow the pandemic [38]. An important development is the utilization of AI methodologies, e.g., for aforementioned deep convolutional neural networks to detect COVID-19 from X-ray images and to identify the development and stage of the disease.

Quickly, several initiatives have come up with stunning new and affordable easy-to-produce CC-P designs, which have often undergone highly sophisticated testing with state-of-the-art equipment to which scientists have access through their labs. The initiatives then typically share their design drawings, CAD files, and testing data adopting open-source licensing approaches, either through formal adoption of open-source hardware (e.g., CERN 2, Apache) and software licenses or informally through statements of intent.

While these initiatives are likely to develop novel foreground CC-IP, they typically build on existing designs and then adopt outbound licensing IP approaches [98]; there is a potential (residual) risk that their designs may actually infringe upon existing CC-IP. As those initiatives usually have limited IP expertise and lack resources to access external legal support (e.g., to hire patent attorneys), it is unlikely that most initiatives conduct freedom to operate analysis before launching their novel CC-P. This lack of due diligence and IP clearance, with the open-source initiatives probably excluding any liabilities and warranties for their designs, could lead any industrial adopter starting to produce an open-source design in volume into trouble, suddenly unwillingly infringing incumbent CC-IP.

The second group of Type 2 entrants includes initiatives that focus on the redesign or new manufacturing approaches for “low-tech” CC-P, i.e., with low technical and manufacturing complexity. Those CC-P include face masks where we have seen numerous initiatives releasing patterns online calling for home production, i.e., crowd-manufacturing. Most of these initiatives might not be seen as highly innovative from an industrial standpoint as they produce fairly mature and “dated” CC-P, which might not be protected anymore by any alive IP. For instance, patents that once protected face mask designs may have long expired.

However, even initiatives focusing on low-tech CC-P clearly innovate by developing novel solutions that go beyond existing CC-P. For instance, different initiatives have started to develop new face shield designs that are optimized for 3-D printing. Those initiatives develop potentially patentable novel CC-IP, while not necessarily infringing incumbent CC-IP as this has probably already expired. Overall, those initiatives may not run into particular infringement risks but are likely to create novel foreground CC-IP. Given that most initiatives adopt open-source licensing approaches, they may not formally seek to protect their novel CC-IP through filing patents. However, we can possibly expect to see some trademark registrations appearing from some of the initiatives that become commercially viable, e.g., those also starting mass 3-D printing production of face shields in Lithuania [99].

#### 3) Scenario 3. IP Considerations for Incumbents

Certain expertise such as vaccine and drug development is so unique to incumbents in CC-S that new entrants are less or almost unlikely to contribute significantly within the short time frames available during a pandemic. Our analysis shows that incumbents can adopt one or more of the following three strategies.

One strategy incumbents can adopt is the development of new technologies and solutions based on their prepandemic technologies and their expertise unique to CC-S. An example is the COVID testing developed by BioFire claimed to be based on its existing technologies (e.g., BioFire Filmarray). Labrador Diagnostics LLC, owned by Fortress Investment backed by SoftBank, however, filed an infringement lawsuit insisting BioFire to stop making the tests as it infringes the claims of two of Labrador's patents [100].

A second strategy that lends itself particularly for prepandemic component suppliers is to establish large-scale manufacturing units to address supply shortages. An example is INEOS, a Chemical giant supplier of one of the key ingredients used in sanitizers, who established mass manufacturing capacities in France to produce about 1 million bottles per month.

The third strategy requires incumbents to collaborate by forming bilateral collaborations (e.g., agreement between Eli Lilly and AbCellera), establish new consortia (e.g., OPENCORONA consortium [74], COVID-19 Therapeutics Accelerator [72]), or join existing networks (e.g., Coalition for Epidemic Preparedness Innovations and Europe's Innovative Medicines Initiative [83]) and thus to share IP among themselves to accelerate the efforts. The consortia are likely to include noncommercial entities such as universities and research centers. Our analysis shows that consortia at the prevention stage focus mainly on vaccine development. Examples include Horizon 2020’s OPENCORONA consortium [74], consortium among Novartis, Bristol Myers Squibb, and GSK [25], and ChAdOx1 consortium involving universities and research centers. For treatment, consortiums are for CC-product (e.g., U.K. Ventilator Challenge consortium [20]) and drugs (e.g., see [83] for several of R&D efforts by incumbents).

Two CC-IP considerations can be identified in the three strategies adopted by incumbents. First, in the efforts by incumbents to accelerate solution development, they may not conduct freedom to operate analysis which takes time. Therefore, they may end up facing infringement lawsuits by other incumbents owning similar CC-IP (e.g., Labrador Diagnostics LLC's lawsuit against BioFire [100]). Second, when prepandemic suppliers establish mass production and manufacturing units, they might generate foreground IP during a pandemic.

## V. Framework for Changing Industrial Organization During Pandemics

Among other activities, research, technology, and innovation efforts are pouring in to support healthcare systems through the development and ramping up the manufacturing of CC-P, such as PPE, diagnostics tests, treatments, ventilators, vaccines, etc. While this calls for existing manufacturers (incumbents) that operate in CC-S to quickly ramp up their production capacities, this also draws various other organizations into CC-S that have not produced CC-P prior to the pandemic. These “new entrants” include large manufacturing firms from related sectors (Type 1 entrants), as well as voluntary initiatives, not-for-profit organizations, scientists, start-ups, and other forms of grassroots initiatives (Type 2 entrants). Open innovation efforts involving incumbents and new entrants become essential to address crisis-critical challenges.

**Fig. 4.**
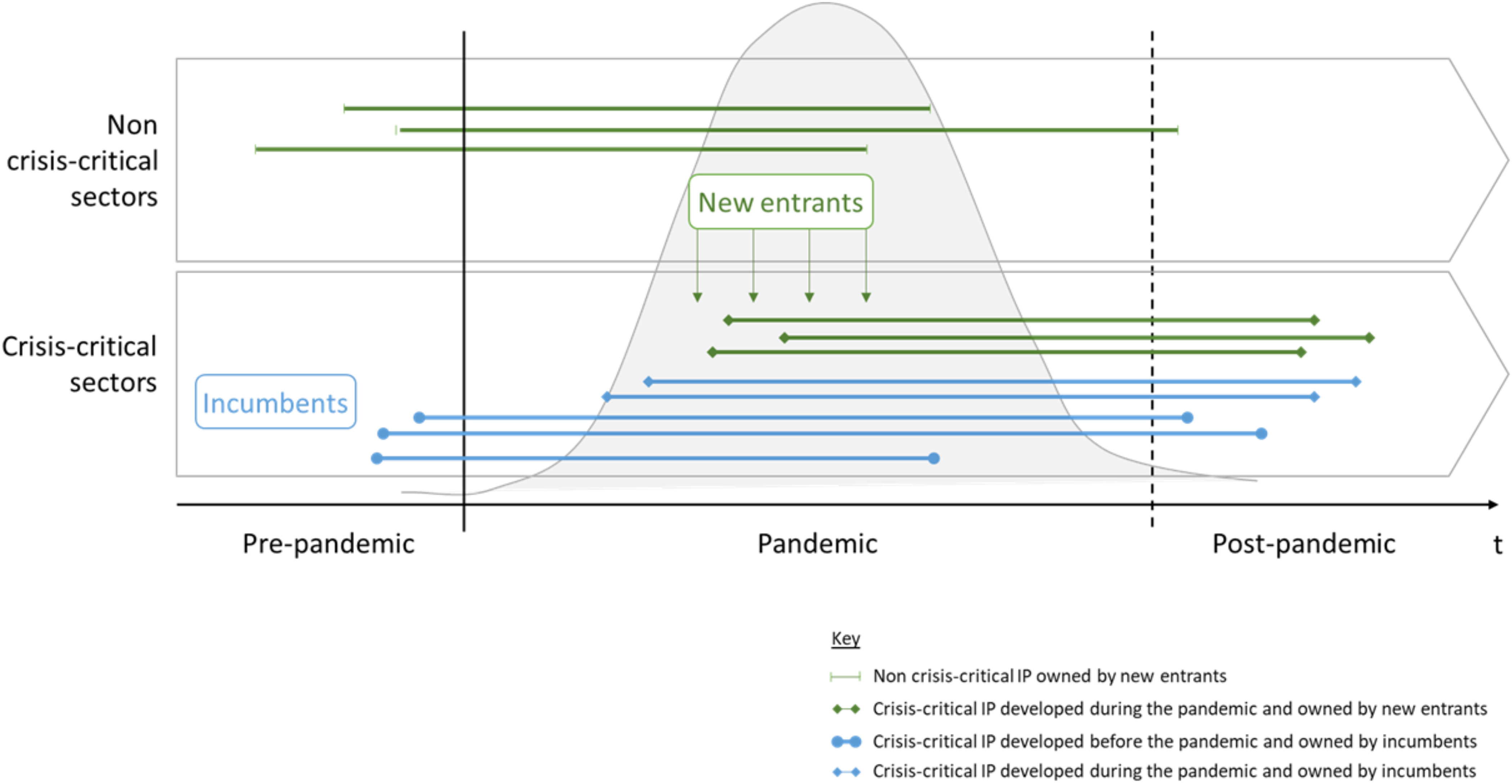
CC-IP during a pandemic.

Incumbents typically own crisis-critical background IP (CC-IP) relevant for the manufacturing of CC-P that new entrants lack; in contrast, when engaging in open innovation for the development and manufacturing of CC-P, new entrants are likely to develop potentially valuable foreground CC-IP during the pandemic. The above scenario considerations together with the identified stakeholders can be summarized in a framework that visualizes how pandemics lead to changing industrial organizations with IP associated challenges (see Fig. 4).

Fig. 4 shows how new entrants are rushing to help the large-scale production of high-tech CC-P (e.g., ventilator production, diagnostic kits), particularly large ones entering CC-S might be more at a risk to infringe active/alive CC-IP owned by incumbents than those entering low-tech CC-P, for which formally relevant IP might have already expired (e.g., face masks) or free alternatives exit. Overall, large firms might be more at risk to become targets for future (i.e., after the pandemic) infringement claims than SMEs and voluntary initiatives. A particular consideration that should be mentioned is that while rushing into CC-P production, large firms may not perceive IP to be that urgent given a current crisis, which could turn out to be difficult in the long run as this could come with considerable IP risks. Incumbent owners of CC-IP might have a lower probability to sue voluntary initiatives, because they cannot claim damages, so could only ask for injunctions, which may cause themselves reputational damages. An exception might be the case of a CC-P developed by a voluntary initiative, which is then produced in large volumes. However, a voluntary initiative giving away its IP open source would not make sufficient money to be a damage claim target. Rather, those manufacturing an open-source design without a clean freedom to operate situation could be targets for infringement claims. One also has to consider that new entrants, whether large manufacturing firms or voluntary initiatives, are likely to develop novel CC-IP during a pandemic, which they could use when continuing to stay in CC-S.

## VI. Policy Responses to Address Pandemic-Related IP Challenges

A pandemic is characterized as a global health crisis that calls for urgent imminent large-scale action by governments, industrial players, as well as a range of other societal actors. Governments take center stage to orchestrate rapid responses. One of their many primary concerns is enabling the mass manufacturing of CC-P, with the demand typically far exceeding the manufacturing capacities available from incumbent players in CC-S. This then calls for other firms to enter CC-S and support the mass manufacturing of CC-P by repurposing manufacturing lines. This leads to situations where incumbents and new entrants find themselves suddenly engaged in new relationships (e.g., U.K. ventilator challenge [28]), possibly even with companies that were competitors before the pandemic.

A pandemic, such as exemplified by the current COVID-10 crisis, leads to wide ranging innovation activities, whether by incumbents or new entrants alone or together (open innovation), being large firms themselves or grassroot initiatives, not-for-profit organizations, or start-ups. While governments’ priority must be to enable the development and mass production of CC-P, they should not forget to address potential IP concerns that incumbents or new entrants may have and think about ways to reduce IP-related risks that could delay the effective mobilization of resources to develop and manufacture CC-P. At least, three approaches have recently been discussed to support the reduction of IP-associated risks.

Compulsory licensing is one legal option that most governments have available. Compulsory licensing is a tool that governments have in those countries that have adopted TRIPS [101]. It allows governments to use IP in crisis situations such as the COVID-19 pandemic. However, compulsory licenses are typically seen as a last resort measure. Compulsory licensing particularly helps governments to access and use CC-IP and thereby reduce IP-associated risks mostly to new entrants, but is usually not favored by incumbents, even though governments typically have to agree to pay a reasonable royalty for accessing their IP. Compulsory licensing measures are a way for governments to access CC-IP, but not necessarily a tool to create innovation incentives, e.g., for developing a vaccine. In the current pandemic, countries that have already used compulsory licensing include Chile, Canada, and Israel [102]–[104].

Another voluntary approach is to call owners of CC-IP (mostly incumbents) to pledge [98], [105], [106] their IP so that new entrants get nonexclusive licenses to use incumbents CC-IP at least for the duration of the pandemic. Examples include firm specific pledges, e.g., by Medtronic [107], AbbVie [75], and Smiths [108], and the Open COVID Pledge^5^^5^[Online]. Available: www.openCOVIDpledge.org
[69], [70] for industrially relevant IP as well as the Wellcome Trust pledge [109] for research publications and data sets. The pledge option, particularly with a time limited license, appears to be more friendly to incumbents while also derisking IP challenges for new entrants. Pledges, such as the Open COVID pledge, also provide license templates that others can use and adjust to their needs.

A third option to reduce IP-associated risks and thus avoid delays in fighting a pandemic are approaches to pool CC-IP, which can then be made available to a restricted group of companies (e.g., a consortium) only or to all interested organizations that want to use that IP. A formal approach for governments would be to facilitate the development of patent pools [110], which have already been used in the pharmaceutical space (e.g., Medicines patent pool).

In addition, we have observed a few more unconventional approaches. To address or avoid IP challenges for instance, very recently, the U.K. announced that it would cover IP-associated risks for manufacturers that engage in ventilator production. This approach appears to be a government-backed IP insurance scheme, which we have not observed elsewhere. Another approach is currently promoted by the European Commission that eliminates IP risks by design. The approach treats the vaccine as THE CC-P and conceptualizes it as a global public good seeking (governmental) investment upfront for its development under the condition that any relevant IP will be freely licensed [118].

**Fig. 5.**
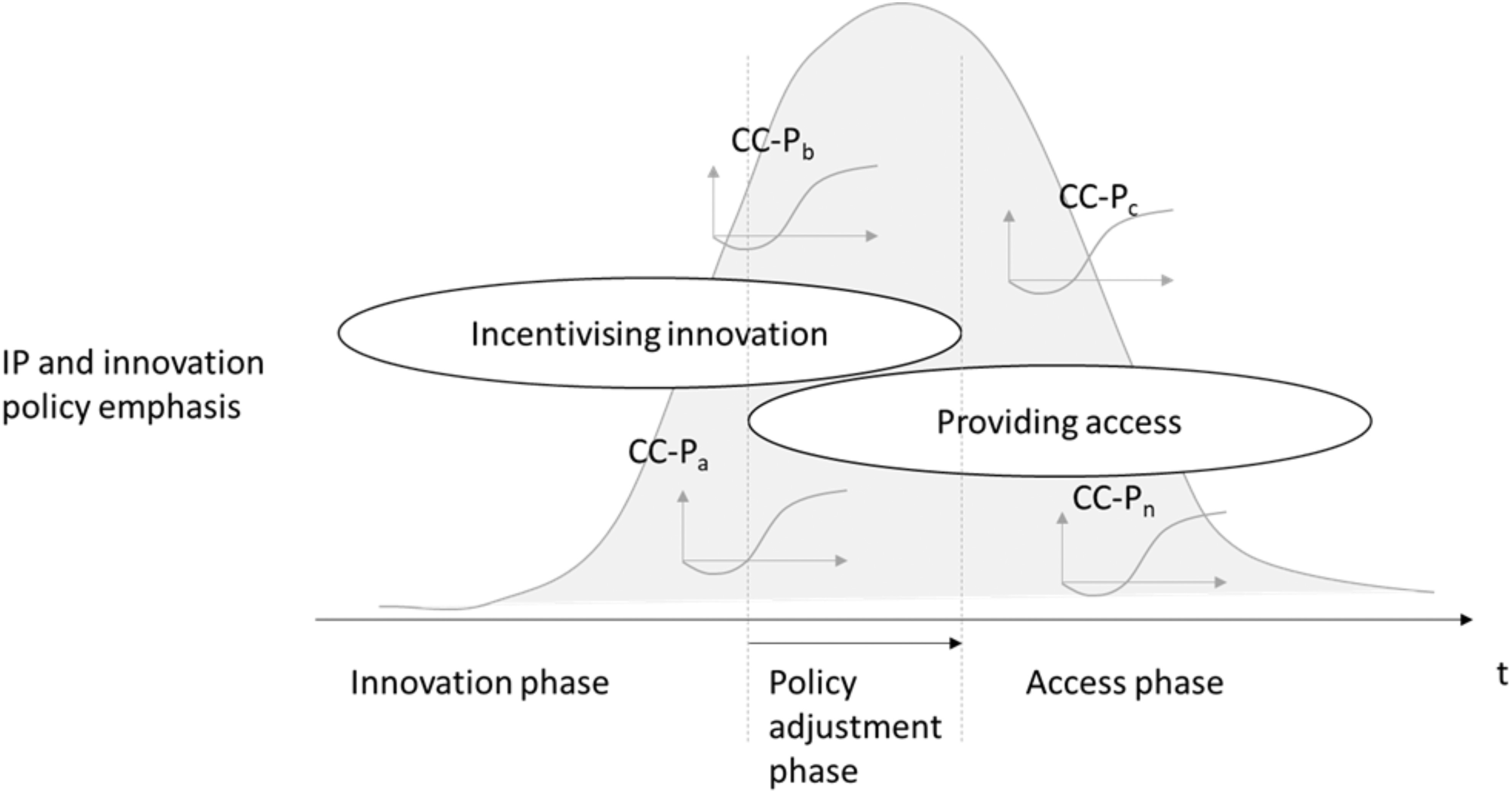
IP policy response principle along a pandemic.

While compulsory licensing has already been enacted by a few countries, the way forward for policy making remains challenging. A guiding principle from an IP and innovation perspective could possibly be summarized in the framework shown in Fig. 5. What appears to be critical during the early stage of a pandemic seems to be policy making for maximizing innovation incentives (as also advocated by WIPO in its recent statement [111]). This goes for the development of CC-P such as PPE in the same way as providing strong incentives for large-scale investments in the development of a vaccine. However, at some point during the pandemic, policy making may have to shift from incentivizing innovation to enabling access to CC-P, particularly the vaccine. To end this pandemic is a global task, and we need to ensure that the vaccine and other lifesaving CC-P, such as ventilators, are distributed to all countries, including the global south. Hence, policy making may have to shift gradually toward encouraging access to CC-IP for CC-P, probably with a point in time to trigger that shift being the availability of a vaccine. However, while this principle may apply to the grand picture, it should not be forgotten that even before the vaccine is available, there are various existing CC-P with associated CC-IP for which policy makers may need to support access with policy measures. Examples are ventilators (for which we have seen some individual firm pledges) and testing kits to allow new entrants scaling up production. Obviously, changing from policy-based incentivizing to facilitating access needs careful consideration. In addition to that, throughout a pandemic, any policy measures that aim to minimize IP-associated risks for key stakeholders (such as the U.K.’s insurance scheme for ventilator manufacturers) are very welcome as any risk can potentially delay the urgent mobilization of innovation capabilities and manufacturing resources.

It should be noted that there is a large body of literature on innovation and IP policy making that can offer potential guidance, reviewing which however was beyond the scope of this article. What seems to be clear from recent observations, however, is that this pandemic—as well as other global challenges—call for large-scale and globally coordinated policy measures (where international bodies such as WIPO could play a more active role), particularly aimed at stipulating open innovation. A range of available policy options to achieve that are offered, for instance, by [119]. As has been shown by firm-level research primarily, IP is important to govern open innovation processes, so it is likely that the global open innovation (eco-)system can be helped by IP and innovation policy making, particularly some forms of licensing agreements. Measures could include, for instance, globally coordinated R&D grants and public innovation procurement schemes for incentivizing innovation together with generous licensing conditions to support access to resulting innovations. Again, we like to stress that these are initial suggestions, based on observations from the past few weeks only.

## VII. Conclusion

From an IP and innovation perspective, this article contributed to the scarce literature about the role of and challenges associated with IP during pandemics. Our findings were derived from analyzing, synthesizing, and interpreting secondary data from the COVID-19 pandemic from two major sources: 1) publicly available documents, such as newspaper articles, industry specific outlets, government reports, and announcements and 2) patent data. Obviously, our findings result only from observations of one ongoing pandemic and thus need to be verified further and interpreted with care.

We find that what makes it difficult for IP to be given its required considerations during the early stage of a pandemic is the enormous sense of urgency, which draws decision makers’ attention to huge and undoubtedly urgent operational challenges. With this article, we hopefully contribute a set of arguments to raise awareness why IP needs to be dealt with earlier rather than later during a pandemic in order to avoid that IP-associated risks delay the mobilization of the resources so urgently needed for the research, development, and mass manufacturing of CC-P. This is particularly important as various responses to the pandemic are somehow technology related, which typically involves IP rights in some form.

This article offered a set of contributions. We summarized IP-related issues currently surfacing during the COVID-19 pandemic in a CC-IP roadmap. We identified four major groups of stakeholders that are mostly concerned with IP considerations. These include governments (and intergovernmental organizations, such as the WHO and WIPO) who are called upon to orchestrate pandemic responses, incumbent manufacturing firms in CC-S, as well as new entrants that enter CC-S to assist incumbents. New entrants include manufacturing firms that did not produce CC-P prior to a pandemic (Type 1 entrants), as well as voluntary grassroot initiatives, start-ups, entrepreneurial scientists, etc. (Type 2 entrants). This article then identified and analyzed three scenarios in which different IP considerations emerge for the different stakeholder groups.

This article provided a terminology that helped to conceptualize IP considerations in times of pandemics or global health crises that call for urgent and large-scale actions from various innovation stakeholders that suddenly find themselves engaged in new relationships that are associated with various IP-associated uncertainties, not the least related to the use and sharing of IP with the particular problem that negotiating licensing agreements is typically time consuming. We also provided a language for policy makers and other decision makers to articulate and discuss IP challenges during pandemics, which might evolve further with specific terms being added gradually or notions being revised as we go along. We proposed a framework that visualizes how industrial organization could change throughout pandemics. That can serve as an analytical framework for others and particularly follow up studies.

Results from our patent analysis show that research and IP protection for coronavirus-related inventions is not new. Patent protection for different forms of coronavirus already exists, but not for the particular coronavirus type SARS-CoV-2 that causes the COVID-19 disease. It appears evident that there is a time lag between outbreaks and the materialization of patents and a number of references to NPL, which shows the urgency of scientists for open data to put the information in the public domain. Any patent analysis is historic, thus limited to existing IP, even with a delay as patent applications get published 18 months after filing. Any patent analysis thus does not capture innovations currently being developed, even though these might result in patent applications, with some of them possibly even having been submitted. Following a systematic identification of CC-P, further specific patent analysis should be conducted to learn more about the owners of IP related to those, which can then, e.g., inform policy makers and help owners of CC-IP to form consortia with others who own complementary IP and identify opportunities for further repurposing of production capacities.

For policy and decision makers, we provide a summary of approaches to address IP concerns during the COVID-19 pandemic, such as compulsory licensing, IP pooling, and IP pledges. We derive initial guiding principles for policy makers toward using IP to maximize innovation incentives for CC-P until these are developed and then shift gradually to use policy measures to facilitate access to these key innovations, such as the vaccine. These should be subject to future scrutiny and needs further work to identify relevant literature from innovation economics. A more advanced IP risk analysis would be helpful to understand the risks for relevant stakeholders during the different pandemic phases, i.e., before/after certain key innovations have been developed, which could then provide relevant input to appropriate policy responses. In fact, currently, we lack systematically collected evidence documenting the extent to which IP issues actually present a barrier or are a perceived possible future problem (risk) and to what extent for different actors. Evidence for this could be created for instance through a survey to those developing and manufacturing CC-P. This would provide a more sound basis for conversations with decision makers about the importance of IP issues during a pandemic.
